# Evaluation of Withania somnifera (L.) Dunal (Ashwagandha) on Physical Performance, Biomarkers of Inflammation, and Muscle Status in Healthy Volunteers: A Randomized, Double-Blind, Placebo-Controlled Study

**DOI:** 10.7759/cureus.68940

**Published:** 2024-09-08

**Authors:** Ashwinikumar Raut, Raakhi Tripathi, Padmaja A Marathe, Dinesh A Uchil, Shubhada Agashe, Nirmala Rege, Ashok B Vaidya

**Affiliations:** 1 Clinical Research and Integrative Medicine Department, Kasturba Health Society, Medical Research Centre, Mumbai, IND; 2 Pharmacology and Therapeutics Department, King Edward Memorial Hospital and Seth Gordhandas Sunderdas Medical College, Mumbai, IND; 3 Clinical and Endocrine Laboratory, Kasturba Health Society, Medical Research Centre, Mumbai, IND; 4 Internal Medicine Department, Kasturba Health Society, Medical Research Centre, Mumbai, IND

**Keywords:** ashwagandha, bicycle ergometer, hand grip dynamometer, muscle mass, sarcopenia

## Abstract

Background: Sarcopenia is associated with chronic inflammation, a sedentary lifestyle, and ageing. However, there exists no drug, which is safe and effective for long-term use. Ashwagandha (*Withaniasomnifera* (L.) Dunal) has the potential to fill this therapeutic gap based on its efficacy and safety profile; hence, the present study was planned to evaluate its effect on inflammatory biomarkers and muscle status in healthy volunteers.

Methodology: A prospective, double-blind, randomized, placebo-controlled clinical study was conducted to evaluate the effects of Ashwagandha extract in healthy volunteers (February 2021 to May 2022) who received either Ashwagandha extract tablets 250 mg or a placebo twice daily for 60 days. The physical performance on a bicycle ergometer, inflammatory/muscle status biomarkers, body composition, reaction time, hemogram, and organ function tests was assessed at baseline, day 30, and day 60.

Results: In the Ashwagandha group, there was a statistically significant (p<0.05) improvement in total distance travelled (Ashwagandha 2.85 ± 0.54 km vs placebo 2.16 ± 0.62 km), average speed achieved (Ashwagandha 25.6 ± 5.7 km/hour vs placebo 22.2 ± 5.48 km/hour) on a bicycle ergometer from the baseline visit (V3) to the last visit (V7) as compared to the placebo group. The observations on hand-grip strength, back-leg press, skeletal muscle mass, and VO_2_ max showed an increasing trend from V3 to V7, whereas the results of the three inflammatory markers (hs-C-reactive protein (CRP) mg/L; IL-6; TNF-alpha ) and the muscle marker (myostatin) revealed a decreasing trend from V3 to V7 in the Ashwagandha group. Ashwagandha extract was found to be safe in healthy volunteers as evidenced by the clinical profile, laboratory investigations, and reaction time test.

Conclusion: Ashwagandha extract supplementation was safe and effective in enhancing physical performance and strengthening muscle mass and could be a potential candidate for treating sarcopenia.

## Introduction

Sarcopenia primarily is the loss of skeletal mass and function associated with the ageing process. However, it is also observed in patients having chronic inflammation, insulin resistance, obesity, protein-energy malnutrition, and physical inactivity [1]. The prevalence of primary sarcopenia in the study population from India was 39.2% [2]. In sarcopenia, muscle loss can be significant enough to cause weakness, increase fall risk, limit physical activities, and adversely affect quality of life. Currently, there are no medications formally approved to treat sarcopenia. Researchers are studying the use of testosterone and growth hormones to help people maintain muscle mass as they age. However, further studies are needed before hormone therapy is recommended to treat sarcopenia. While these agents have shown some positive effects on muscle strength and mass, their use is limited owing to adverse effects, such as an increase in the risk of prostate cancer in men, virilization in women, and an overall high risk of cardiovascular events [3]. Hence, there is an unmet need to search for safer alternatives for sarcopenia.

As per Ayurveda, the term ‘mamsakshaya’ is derived from two words ‘mamsa’, meaning ‘muscle’, and ‘kshaya’ meaning ‘loss’ [4], which can be considered equivalent to sarcopenia. Different treatment modalities have been practiced for mamsakshaya, such as dietary substances, physical activity, lifestyle measures, and medicinal plants with *Rasayana* (rejuvenative) properties. 'Rasayana' can be defined as ‘Labhopayohi shastanam rasadinam rasayanam’, which means the modality of Rasayana provides optimum levels of excellent body tissues [5]. Thus, Rasayana is a modality that can improve body defence mechanisms, improve cognitive abilities, prevent ageing, and boost vitality [6]. Ashwagandha (Withania somnifera) is acknowledged as one such plant having 'Rasayana' properties. It is ascribed to the properties of ‘balya’ (enhancing strength and immunity)and ‘mamsavivardhan’ (enhancing muscle mass and function) [7,8].

Our previous study demonstrated that Ashwagandha tested in healthy volunteers was tolerated at high doses and increased muscle activity [9]. Another study conducted on healthy volunteers has also shown improvement in physical performance [10]. In a study by Pandey et al., treatment with Ashwagandha improved weakness, fatigue, and quality of life in perimenopausal and postmenopausal women [11].

The primary objectives of the present study were to evaluate the effects of Ashwagandha on biomarkers of inflammation and muscle status in response to exercise. The secondary objectives of the study were to evaluate the effects of Ashwagandha on physical performance and body composition. In addition, the safety of Ashwagandha was assessed by hematological investigations and organ function tests. Since Ashwagandha is known to cause sedation, reaction time was also assessed to evaluate alertness [12].

## Materials and methods

This was a prospective, double-blind, randomized, placebo-controlled clinical study conducted in the Department of Pharmacology and Therapeutics, King Edward Memorial Hospital and Seth Gordhandas Sunderdas Medical College, Mumbai, after obtaining Institutional Ethics Committee approval (project No EC/OA-159/2018; approval dated 9/7/2019) and registered with Clinical Trials Registry India (CTRI/2019/07/020278) from February 2021 to May 2022. It was carried out in accordance with the Declaration of Helsinki and National Ethical Guidelines for Biomedical and Health Research Involving Human Healthy Volunteers issued by ICMR (2017) [13].

The study population targeted 60 healthy individuals aged between 18 and 35 years of either gender, a body mass index (BMI) range within 18-30 kg/m^2^, non-smoker, non-alcoholic having a normal physical examination, hematological and biochemical laboratory parameters, and with no history of lower limb or upper limb surgery in the past six months. Those healthy volunteers who were willing to abstain from unaccustomed strenuous physical activity and maintain a constant diet pattern throughout the duration of the study starting at least one week before the study providing written informed consent were selected. Participants were excluded if they had a history of allergy/intolerance to herbal medication, concomitant intake of medications (from traditional systems), any chronic medication, vitamins, antioxidants, oral contraceptives, anabolic steroids, protein supplements, nutritional supplements, and recreational/addicting drugs.

The therapeutic dosage range of Ashwagandha is 3-5 g of coarsely pulverized roots [14]. Thus, the tablets Agewel^TM^ (250 mg/tablet) procured from Pharmanza Herbal Pvt. Ltd., Gujarat, contained a hydroalcoholic extract of Ashwagandha roots and were standardized for total withanolides concentration as 1.5% and the daily dose was 500 mg. Thirty-six Ashwagandha tablets were packed in one container with an air-tight lid and stored at room temperature, between 24 and 32°C [15,16].

The healthy volunteers were randomized in a 1:1 ratio by a computer-generated code system for allocation to treatment groups by the co-investigator. The healthy volunteers received either a tablet of Ashwagandha (WS) extract (250 mg) or an identical placebo (P; 250 mg (dextrin)), twice a day (12 hourly) for 60 days. These tablets were dispensed in coded bottles as the study was double-blind by the pharmacist.

**Figure 1:**
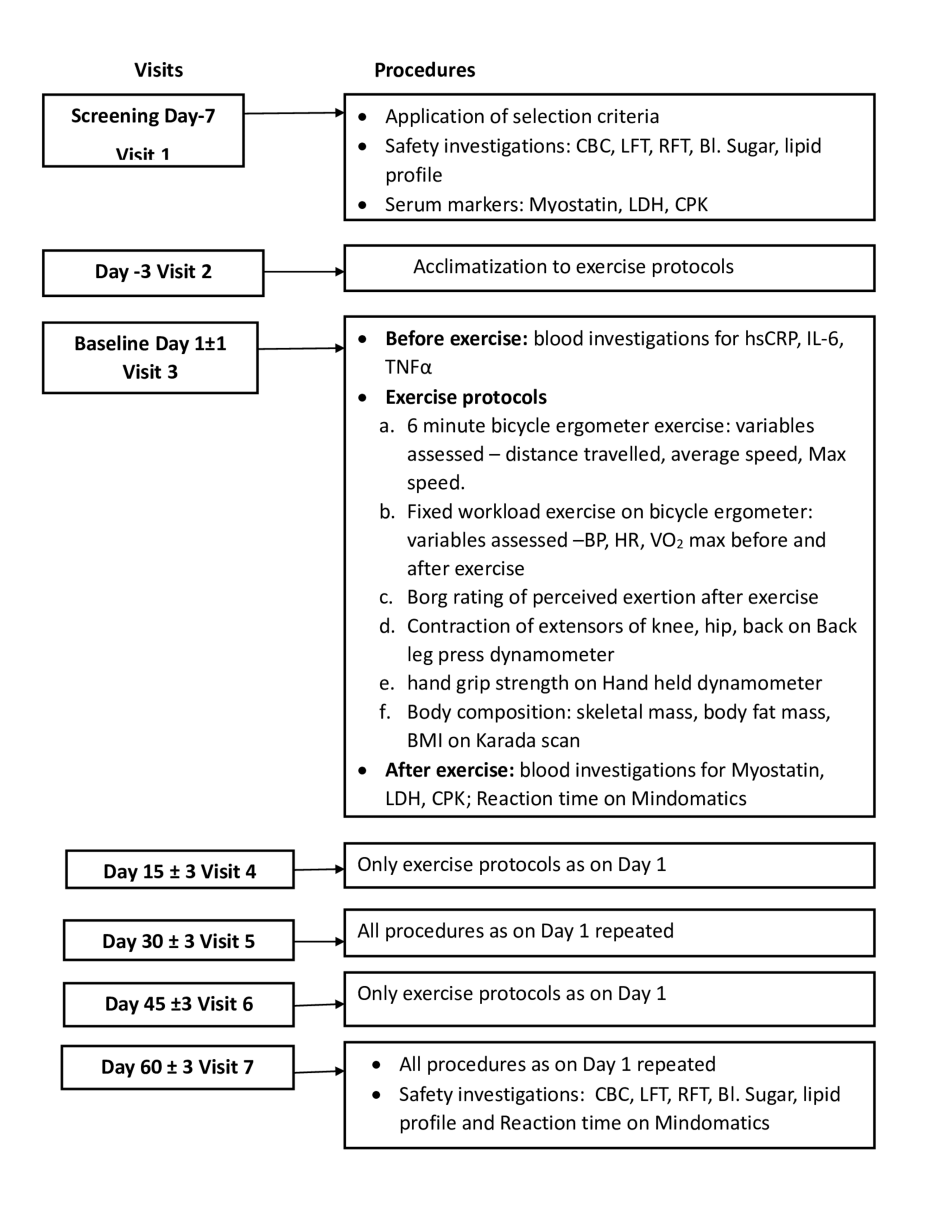
Visit-wise study procedures for the volunteers CPK: Creatine phosphokinase, LDH: Creatine phosphokinase, IL-6: Interleukin 6, hs-CRP: High-sensitivity C-reactive protein, TNF-α: Tumor necrosis factor-alpha

The total duration of participation was two months comprising of a screening visit (day seven) (first visit): thereafter, on day three visit two), day one (third visit), day 15 (fourth visit), day 30 (fifth visit), day 45 (sixth visit), and day 60 (seventh visit). A detailed medical history and physical examination were carried out. The healthy volunteers were assessed at each visit for a six-minute bicycle ergometer test, fixed workload exercise on a cycle ergometer, back-leg press dynamometer, hand-held dynamometer, and for body composition using the Karada scan. Serum biomarkers (serum CPK, serum LDH, serum myostatin) were assessed on day seven (at the screening to indicate pre-exercise values), day one, day 30, and day 60 post-exercise. The inflammatory biomarkers (hs-CRP, IL-6, TNF-α) were assessed on days one, 30, and 60 before exercise. Laboratory investigations (complete blood count, blood sugar level, liver and renal function tests, lipid profile) were done at screening and day 60. The study team regularly enquired about the adverse events encountered during the study period and reaction time was also noted on days one and 60. The visit-wise procedures are depicted in Figure 1.

The physical performance tests are described as follows:

(1) Six-minute cycle ergometer exercise [10,17,18]: The volunteer rested for 10 minutes, the pulse oximeter unit was attached, and resting (average of three BP readings in sitting position) blood pressure (BP) and heart rate (HR) were recorded. The volunteer was instructed to peddle the bicycle continuously for six minutes at no set pace at his/her full effort. The resistance on the bicycle ergometer was maintained at 75 watts during the activity. The distance travelled (in km), maximum speed, and average speed attained (in km/hour) were recorded. To achieve hemodynamic stabilization following this test, 10 minutes of rest was provided before the commencement of the next test.

(2) Fixed workload exercise on bicycle ergometer [19]: After a 10-minute break following the six-minute cycle ergometer exercise, the pulse oximeter was kept connected to the volunteer during the exercise to measure BP and HR. VO_2_ max was calculated indirectly using three-minute workloads that were designed to raise HR between 110 bpm and HR that is near 85% of age-predicted HRmax. Workload at 25 watts and a pedaling rate of 60 rpm was kept for the first three minutes. After the third minute, based on HR, the subsequent second load was kept. The third and fourth workloads (if required) were set by adding a workload of 25 watts to the previous workload. HR was measured during minutes two and three at each workload. If these HR were not within 5 bpm, then HR was recorded at four minutes. VO_2_ max was later calculated using the YMCA cycle ergometer submaximal test equation: ((workload/body mass in kg) x 10.8) + 3.5 + 3.5.

(3) Borg rating of perceived exertion after exercise [20]: This method was used to gauge the intensity of physical exercise. The rating ranged from 6 to 20, where 6 meant 'no exertion at all' and 20 meant 'maximal exertion.'

(4) Back-leg press dynamometer [21]: The back-leg press dynamometer test was conducted following a 10-minute break. Subsequently, the handle was positioned at the height of the intra-articular space of the knee joint. To complete the test, the volunteer is instructed to stand on the platform with their knees and hips slightly bent and their lower back in a suitable lordotic curve. The volunteer was asked to lift the handle in a vertical direction by providing continuous isometric contractions of the extensors of the knees, hips, and lower back while holding the handle. There were 30-second breaks in between each of the three trials.

(5) Hand grip strength by hand-held dynamometer [22]: The dominant hand's grip strength was assessed using a hand-held dynamometer. The volunteer was instructed to grasp/squeeze the handle tightly. Three readings were taken with a gap of one minute in between the readings, and the average of these three readings was noted.

(6) Body composition: Skeletal muscle mass, body fat mass, and BMI were assessed using a body composition analyzer with the Karada scan (MC-780MA).

All visit procedures were conducted in the Neuropharmacology Laboratory, Department of Pharmacology and Therapeutics. All the volunteers were enquired about the occurrences of adverse events throughout the study. The record was kept of examination of vitals for all volunteers. A complete hemogram using the Diatron kit, fasting blood glucose level, lipid profile (serum cholesterol, triglycerides), and organ function tests (serum glutamic-oxaloacetic transaminase (SGOT), serum glutamic-pyruvic transaminase (SGPT) and blood urea nitrogen (BUN), S. creatinine) were carried out using the Erba XL system pack at the baseline visit and day 60.

To assess the effect of Ashwagandha on the level of alertness, and reaction time on Mindomatics™ (consisting of a specially designed response keyboard and Mindomatics software) was noted using software (M/s Sristek, Hyderabad). The volunteer was made to sit in a quiet room. After clicking on the start button, a picture of a boy/girl appeared in the center of the monitor screen, 10 times each in a random sequence over the duration of the test period. The volunteer was instructed to press with the left index finger on the response keyboard as quickly as possible, matching the monitor picture. Each test ended after 60 seconds. This technique aided in analyzing the choice discrimination test. To assess the digit picture substitution, 1-9 numbers were displayed in the upper panel of the screen, each with a target image above it. The volunteer was instructed to pay close attention and recall the image that goes with each of these digits. The volunteer had to press the corresponding digit as quickly as possible on the response box when the target picture appeared. Each test lasted for 90 seconds. The reaction time (in milliseconds), total number of attempts, and correct and wrong attempts were noted [23,24].

Statistical analysis

The statistical analyses were done using Statistical Product and Service Solutions (SPSS, version 21; IBM SPSS Statistics for Windows, Armonk, NY) software. Qualitative data were expressed as frequencies and percentages, while the quantitative data were expressed as mean ± standard deviation (SD). The normality of the data was assessed using the Shapiro-Wilk test. Appropriate statistical tests with post-hoc tests were employed to compare the groups. Based on the normality, an unpaired t-test for parametric data/Wilcoxon signed rank test (Mann-Whitney test) for non-parametric data was employed to compare data between the two groups. We employed repeated measures ANOVA/Friedman test to analyze the data at multiple time points. One-way ANOVA/Kruskal-Wallis test was used to compare the data in three or more groups. The chi-square/Fischer's exact test was used to compare the frequency of adverse reactions between the two groups. The level of significance was set as p<0.05. The primary objective of the present study was to evaluate the effects of Ashwagandha on biomarkers of inflammation, and no data were available in the literature to estimate the effect size; hence, this was planned as a pilot study, and no formal sample size calculation was done. As it was a proof-of-concept study, 30 participants per arm was considered optimum to start with.

## Results

**Figure 2:**
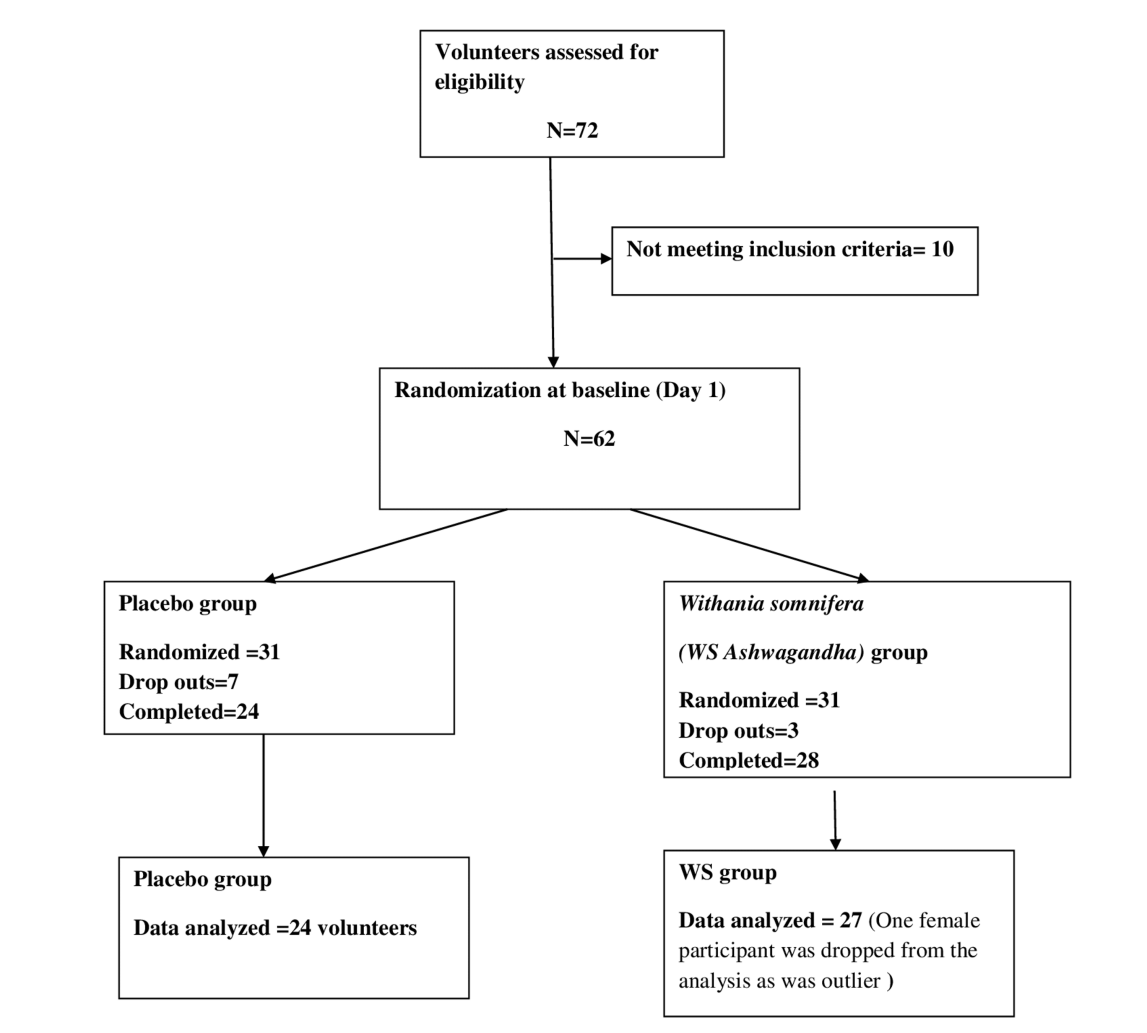
CONSORT flow representation of healthy volunteer enrolment and allocation CONSORT: Consolidated Standards of Reporting Trials

Seventy-two healthy volunteers were assessed for eligibility. Of these, 10 healthy volunteers failed to meet the eligibility criteria. Out of 62 volunteers recruited, 31 volunteers were randomized equally into two groups to receive a placebo and Ashwagandha extract. The study was completed by 24 volunteers who received a placebo, while 28 volunteers completed the study in the ashwagandha group. There were all male volunteers in the placebo group, while in the Ashwagandha group, there were 27 male and one female volunteer. The data received from this female volunteer were excluded; thus, data from 27 volunteers was analyzed in the Ashwagandha group (Figure 2).

The demographic profile of healthy volunteers in the Ashwagandha extract (WS) group and placebo group is depicted in Table 1; nevertheless, the unpaired T-test revealed no statistically significant difference between the two groups (p>0.05).

**Table 1:** Demographic profile of healthy volunteers in both placebo and Ashwagandha group All variables are expressed as mean ± SD, no statistical difference detected with the unpaired t-test

Sr no	Variable	Placebo (n = 24)	Ashwagandha (WS; n = 27)
1	Age (in years)	26.76 ± 2.1	27.11 ± 1.8
2	Height (in meters)	1.7 ± 0.08	1.7 ± 0.05
3	Weight (in kg)	69.31 ± 8.06	69.38 ± 7.7
4	BMI (kg/m^2^)	23.4 ± 3.29	23.9 ± 2.73
5	Axillary Temperature (F)	36.39 ± 0.2	36.38 ± 0.2
6	Systolic Blood Pressure (mmHg)	123.4 ± 5.8	121.7 ± 6.9
7	Diastolic Blood Pressure (mmHg)	76.6 ± 8.02	77.4 ± 7.5

Table 2 tabulates the comparison of the efficacy variables based on the physical performance of the placebo and Ashwagandha extract group on day one, day 15, day 30, day 45, and day 60. The total distance on the bicycle ergometer was significantly higher (p=0.001) on days 30, 45, and 60 compared to that on day one. On comparing the effects of Ashwagandha extract and placebo on variables of physical performance at day 60, it was found that the average speed and total distance were statistically higher in the ashwagandha extract group; a statistically higher SBP after exercise (p=0.045) and Borg rating scale (p=0.015) were noted in this group. However, no statistical significance was noted in other physical parameters of assessment. VO2 max (in mL/kg/min) was compared between the placebo and Ashwagandha extract (WS)-group at day one (56.18 (35.35-62.99) versus 33.43 (24.38-51.47)) with p=0.03 and day 60 (42.06 (33.66-67.36) versus 37.24 (32-47.14)) with p=0.21 on the Mann-Whitney U test. Further, there was no statistical significance when a comparison was made between day one and day 60 within each study group (p>0.05).

**Table 2:** Comparison of physical performance variables between the placebo (P) and Ashwagandha extract (WS) groups WS=Ashwagandha extract, P=placebo, bpm=beats per minute * Represents a p-value<0.05 on the repeated measures ANOVA test where a comparison of the placebo arm (within the group) is carried compared to baseline visit # Represents a p-value<0.05 on the repeated measures ANOVA test where a comparison of the Ashwagandha extract arm (within the group) is carried with baseline visits; $ represents p<0.05 on comparison between the placebo and Ashwagandha extract arms (between the groups) for that particular visit on the independent T-test @Borg rating ranged from 6 to 20, where 6 meant "no exertion at all" and 20 meant "maximal exertion"

No.	Variable	Day 1 Mean ± SD		Day 15 Mean ± SD		Day 30 Mean ± SD		Day 45 Mean ± SD		Day 60 Mean ± SD	
		P n=24	WS n=27	P n=24	WS n=27	P n=24	WS n=27	P n=24	WS n=27	P n=24	WS n=27
1	HR before bicycle ergometer exercise (bpm)	75.48 ± 8.4	79.56 ± 9.1	77.92 ± 8.4	77.89 ± 8.2	76.36 ±9.5	78.48 ± 8.59	76.68 ± 8.3	78.11 ± 8.9	75.16 ± 8.6	76.41 ± 8.7
2	SBP before bicycle ergometer exercise (mmHg)	121.6 ± 12.5	126 ± 12.2	120.5 ± 10.9	126.1 ± 12.6	119.6 ± 9.63	124.3 ± 13.8	119.9 ± 10.9	126.19 ± 10.6	120.2 ± 11.35	124.78 ± 12.1
3	DBP before bicycle ergometer exercise (mmHg)	75.68 ± 9.8	78.63 ± 10.7	75.44 ± 9.3	79.11 ± 11.46	73.68 ±10.3	77.74 ± 10.1	73.56 ± 8.8	76.78 ± 10.3	75.36 ± 5.05	77.56 ± 4.9
4	HR after bicycle ergometer exercise (bpm)	110.36 ± 14.1	114.81 ± 16.04	110.44 ± 9.9	115.93± 13.3	109.4 ±11.8	114.37 ± 14.3	111 ± 11.4	116.89 ± 14.9	108.56 ± 10.8	113.85 ± 12.6
5	SBP after bicycle ergometer exercise (mmHg)	140.32 ± 14.8	145.89 ± 12.5	140.96 ± 13.1	149.63± 15.8	137.64±11.9	143.85 ± 13.8	142.96 ± 11.5	145.93 ± 15	138.56± 9.5	145.81 ± 14.8^$^
6	DBP after bicycle ergometer exercise (mmHg)	81.88 ± 11.9	80.89 ± 12.5	85.04 ± 9.6	79.26 ± 12.6	84.52 ± 8.8	76.48 ± 10.1	82.76 ± 8.2	78.19 ± 10.8	79.96 ± 7.2	79.07 ± 10.1
7	Total distance on bicycle ergometer (km)	2.08 ± 0.6	2.07 ± 0.7	2.08 ± 0.6	2.15 ± 0.6	2.28 ± 0.5	2.83 ± 0.6^#^	2.26 ± 0.6	3.01± 0.7^#^	2.16 ± 0.62	2.85 ± 0.54^#$^
8	Maximum speed on a bicycle ergometer (km/hr)	26.9 ± 9.13	27.5 ± 8.7	27.8 ± 9.35	28.4 ± 9.5	28.6 ± 6.07	29.4 ± 8.9	28.6 ± 8.2	29.8 ± 8.6	28.8 ± 7.7	29.8 ± 7.6
9	Average speed on a bicycle ergometer (km/hr)	20.3 ± 5.3	22.6 ± 13.6	20.9 ± 5.6	22.5 ± 6.3	22.3 ± 5.1	23.3 ± 6.7	22.5 ± 5.6	24.8 ± 6.8	22.2 ± 5.48	25.6 ± 5.7^$^
10	Fixed workload HR at rest (bpm)	78.64 ± 14.2	79.37 ± 9.1	78.24 ± 8.7	77.8 ± 8.2	77.24 ± 8.96	78.07 ± 9.7	76.6 ± 8.6	78.11 ± 8.9	75.76 ± 8.4	76.7 ± 8.4
11	Fixed workload HR at end (bpm)	112.7 ± 18.4	115.7 ± 15.7	110.28 ± 9.9	115.3 ± 13.5	110 ± 12	114.52 ± 14.06	111.6 ± 11.03	116.96 ± 14.7	110.44± 10.5	113.85 ± 12.6
12	Back-leg press dynamometer(muscle force in kg)	45.9 ± 19.8	45.6 ± 17	45.5 ± 14.2	45.7 ±15.2	43.2 ± 13.2	46.9 ± 18.4	43.6 ± 12.9	46.8 ± 20.5	43.9 ± 11.3	47.12± 16.3
13	Hand grip strength(muscle force in kg)	35.2 ± 10.7	34.3 ± 7.7	34.06 ± 9.6	35.8 ± 6.97	34.1 ± 9.1	35.3 ± 6.3	34.1 ± 8.6	36.12 ± 6.4	34.68 ± 8.6	36.4 ± 6.6
14	Body composition skeletal muscle mass (%)	75.8 ± 6.5	74.83 ± 3.5	75.6 ± 5.7	75.1± 3.91	75.4 ± 5.1	75.6 ± 3.67	75.4 ± 5.8	75.3 ± 4.09	75.7 ± 5.1	75.84 ± 3.89
15	Body composition body fat mass (%)	19.8 ± 6.4	21.1 ± 3.7	20.4 ± 6.6	20.8 ± 4.13	20.4 ± 5.4	21.4 ± 3.88	20.4 ± 6.1	21.6 ± 4.34	20.1 ± 5.4	21 ± 4.13
16	Body composition BMI (kg/m^2^)	23.4 ± 3.4	23.9 ± 2.7	23.7 ± 3.4	24.1 ± 2.7	23.5 ± 3.3	24.1 ± 2.7	23.6 ± 3.4	24.1 ± 2.6	23.6 ± 3.4	24.3 ± 2.64
17	Borg Rating^@^	13.2 ± 1.5	13.63 ± 1.9	12.6 ± 1.4	13.11 ± 1.4	12.24 ± 1.3*	13.04 ± 1.4	12.64 ± 1.2	13.19 ± 1.4	12.16 ± 1.25*	13.37 ± 2.1^$^

Assessment of biomarkers for inflammation and muscle status in healthy volunteers is represented in Table 3. In the Ashwagandha extract group by day 60, the inflammatory biomarkers viz. hs-CRP, interleukin-6 (a significant decrease on day 30 compared to day one), and TNF-α and muscle biomarker serum myostatin showed a decreasing trend. However, a significant increase was noted in the serum CPK on day 60 compared to day seven. Meanwhile, in the placebo group, the markers increased, especially for hs-CRP (a significant increase on day 60 compared to day one), serum CPK (a significant increase on day 60 compared to day seven), and lactate dehydrogenase (a significant increase on day 60 compared to day seven). A decreasing trend was noted gradually at every subsequent visit in serum myostatin in the Ashwagandha extract (WS) group by day 60; however, no statistical significance was noted.

**Table 3:** Assessment of biomarkers of inflammation and muscle status in healthy volunteers in the placebo and Ashwagandha groups WS=Ashwagandha extract, P=placebo * Represents a p-value<0.05 on the Friedman test where a comparison of the placebo arm (within the group) is carried with a baseline visit; # Represents a p-value<0.05 on the Friedman test where a comparison of the Ashwagandha extract arm (within the group) is carried with a baseline visit; $ represents p<0.05 on a comparison between the Ashwagandha extract and placebo groups (between the groups) for that particular visit on the Mann-Whitney U test Serum CPK, serum LDH, serum myostatin: assessed on day seven (screening visit), day one, day 30, and day 60 post-exercise; day seven indicated the pre-exercise values. Serum hs-CRP, serum IL-6, and serum TNF-α: assessed on days one, 30, and 60 before exercise

No.	Variable	Day -7 (Screening) Mean ± SD		Day 1 Mean ± SD		Day 30 Mean ± SD		Day 60 Mean ± SD	
		P n=24	WS n=27	P n=24	WS n=27	P n=24	WS n=27	P n=24	WS n=27
1	High-sensitivity C-reactive protein (mg/L)			1.08 ± 1.02	2.28 ± 3.85	2.08 ± 2.54	2.06 ± 2.55	4.6 ± 9.2*	1.76 ± 2.23
2	Interleukin-6 (pg/mL)			7.72 ± 8.91	14.98 ± 21.3	5.74 ± 4.81	6.19 ± 4.89^#^	5.65 ± 4.25	7.2 ± 6.45
3	Tumour necrosis factor-α (pg/mL)			12.6 ± 13.78	12.43 ± 11.6	10.44 ± 6.29	9.25 ± 5.05	10.74 ± 9.72	8.05 ± 2.65
4	Serum myostatin (ng/mL)	5.64 ± 8.44	6.81 ± 8.09	5.74 ± 8.73	6.5 ± 8.58	4.89 ± 6.87	6.37 ± 8.29	5.47 ± 8.39	5.9 ± 7.6
5	Serum creatine phosphokinase (U/L)	132.27 ± 23.78	125.35 ± 31	137.91 ± 34.12	135.2 ± 25.98	142.24 ± 34.04	131.02 ± 39.21	160.59 ± 45.99*	145.41 ± 22.68#
6	Lactate dehydrogenase (IU/L)	367.28 ± 95.39	390.55 ± 96.77	362.76 ± 84.59	396.9 ± 67.91	354.77 ± 75.72	391.76 ± 79.6	412.93 ± 136.12*	408.52 ± 109.02

No significant difference was noted among the hematological and serological variables. Although a significant change was seen in total cholesterol for both groups (refer to Table 4), no serious adverse events were reported in any of the groups. The Ashwagandha extract group showed a statistically significant reduction in reaction time in the digit substitution test, and the same trend was seen in the choice discrimination test. This indicates improved alertness and no adverse effect on cognitive functions (refer to Table 5).

**Table 4:** Safety investigation results in the participants of the placebo and WS groups WS=Ashwagandha, P=placebo *Represents p<0.05 between day seven and day 60 of the placebo group on the paired T-test; #Represents p<0.05 between day seven and day 60 of the WS group on the paired T-test; $represents p<0.05 between the placebo and WS groups on the unpaired T-test

No.	Variable	Day -7 (screening) Mean ± SD		Day 60 Mean ± SD	
		P n=24	WS n=27	P n=24	WS n=27
1	RBC (million/mm^3^)	5.01 ± 0.5	4.79 ± 0.5	9.84 ± 13.72	7.56 ± 10.35
2	WBC (million/mm^3^)	6912 ± 1847.9	7672.2 ± 1797.5	7059.6 ± 1324.02	6584.81 ± 1645.38
3	Polymorphs (%)	60.96 ± 4.97	63.85 ± 5.36	62.8 ± 4.85	63.7 ± 4.61
4	Lymphocytes (%)	38.48 ± 5.29	35.37 ±4.8^$^	36.92 ± 3.93	35.04 ± 4.75
5	Monocytes (%)	0.12 ± 0.44	0.15 ± 0.36	0.28 ± 0.61	0.3 ± 0.87
6	Fasting blood sugar [FBS] (mg/dL)	88.1 ± 7.95	90.69 ± 6.98	91.06 ±9.5	92.4 ± 7.87
7	Serum cholesterol(mg/dL)	113.5 ± 60.96	108.9 ± 65.82	172.04 ± 33.7*	178.24 ± 47.4^#^
8	Serum triglyceride(mg/dL)	99.18 ± 29.26	95.57 ± 29.61	106.3 ± 48.17	111.47± 67.85
9	SGPT(IU/L)	21.6 ± 11.73	26.9 ± 11.8	21.61 ± 11.56	26.4± 11.89
10	SGOT(IU/L)	23.5 ± 5.8	25.46 ± 5.98	23.3 ± 5.4	26.8 ± 8.34
11	Serum creatinine (mg/dL)	0.95 ± 0.17	0.96 ± 0.16	0.98 ± 0.2	0.89 ± 0.22
12	Blood urea nitrogen(mg/dL)	10.41 ± 2.42	10.84 ± 3.28	12.04 ± 3.87	12.53 ± 6.01

**Table 5:** Comparison of the results on Mindomatics for the participants in the placebo and Ashwagandha extract groups WS=Ashwagandha extract # Represents p<0.05 on the Wilcoxon signed-rank test comparing day one and day 60 in the Ashwagandha extract arm.

Sr. no	Mindomatics evaluation						
		Placebo			Ashwagandha (WS)		
1	Choice Discrimination Test (Reaction Time in milliseconds)	Day 1	Day 60	P value	Day 1	Day 60	P value
		Mean ± SD	Mean ± SD		Mean ± SD	Mean ± SD	
		609.12 ± 84.17	622.60 ± 85.15	0.457	731.74 ± 753.19	599.33 ± 75.05	0.657
	Digit picture substitution test (Reaction time in milliseconds)	1941.52 ± 312.32	1866.36 ± 237.87	0.086	2022.07 ± 417.8	1821.74 ± 218.99^#^	0.001

## Discussion

About 70-80% of the population around the globe relies on traditional systems of medicine, according to a report by the WHO [25]. The adverse effects of modern medicines, their cost, the inability to offer a cure for many chronic conditions, and the problem of drug resistance have boosted public interest in traditional systems of medicine such as Ayurveda, which is a science of life that emphasizes personalized treatment and a holistic approach to health [26]. This study was conceptualized on the principles of reverse pharmacology, which facilitates the translation of experience-based traditional wisdom into experiments-based clinical evidence [27].

The results of this study demonstrated that Ashwagandha hydroalcoholic root extract in a dose of 250 mg twice a day over two months significantly (p<0.05) and gradually improved the total distance travelled (Ashwagandha 2.85 ± 0.54 km vs placebo 2.16 ± 0.62 km) and the average speed achieved (Ashwagandha 25.6 ± 5.7 km/hour vs placebo22.2 ± 5.48 km/hour) on a bicycle ergometer from the baseline visit (V3) to the last visit (V7) as compared to the placebo group. The observations on hand-grip strength (force in kg: 34.3 ± 7.7 to 36.4 ± 6.6), back-leg press (force in kg: 45.6 ± 17 to 47.12 ± 16.3), skeletal muscle mass (%: 74.83 ± 3.5 to 75.84 ± 3.89), showed an increasing trend from baseline V3 to last visit V7. Meanwhile, the three inflammatory markers (hs-CRP mg/L: V3 of 2.28 ± 3.85 to V7 or 1.76 ± 2.23; IL-6 pg/mL: V3 of 14.98 ± 21.3 to V7 of 7.2 ± 6.45; TNF-alpha pg/mL: V3 of 12.43 ± 11.6 to V7 of 8.05 ± 2.65), as well as the muscle marker (myostatin ng/mL) revealed a decreasing trend from V3 (6.5 ± 8.58) to V5 (6.37 ± 8.29) and V7 (5.9 ± 7.6) in the Ashwagandha group. Ashwagandha extract was found to be safe in healthy volunteers, as evident from the clinical profile, laboratory investigations, and reaction time test.

The study results showed that the maximum speed on the bicycle ergometer gradually increased for both groups at subsequent follow-up visits. The change in the maximum speed attained in the Ashwagandha extract group was greater than that of the placebo group, though not statistically significant. The increasing trend in the placebo group over time may be attributed to healthy volunteers becoming accustomed to the study procedure on consecutive visits. However, in the Ashwagandha extract group, in addition to this customization, there is a concomitant increment in the average speed and distance travelled, which was not evident in the placebo group. Although the Ashwagandha extract group noticed a statistically significant rise in SBP immediately following exercise, the values remained within the normal range and were not clinically significant.

The observations in the back leg press dynamometer as well as hand-held dynamometer tests by the Ashwagandha extract group also revealed an increase in values after day 15. Although between the two groups, there was no statistically significant difference, the gradual improvement in both tests suggests improvement in muscle strength with Ashwagandha extract. The current study assessed the VO_2_ max, which highlights long-term aerobic and cardiovascular endurance. In contrast to the placebo, which showed a decline in VO_2_ max values from 56.18 mL/kg/min to 42.06 mL/kg/min, Ashwagandha extract demonstrated an improvement from 33.43 mL/kg/min to 37.24 mL/kg/min. These results suggest that Ashwagandha extract may help improve cardiovascular endurance, though not statistically significant. The Ashwagandha extract group had a significantly higher Borg rating scale than the placebo group, which may be attributed to an increase in the overall distance travelled by the group.

The study also showed a gradual rise in body composition of skeletal muscle measurements and total BMI increment without an increase in total fat mass in the Ashwagandha extract group. These findings indicate a positive trend of Ashwagandha extract towards improvement in muscle function.

The study's findings corroborated those of Sandhu et al., who highlighted that Ashwagandha extract administration for eight weeks significantly increased barbell velocity, muscle power, and VO_2_ max and lowered resting SBP [28]. In addition, the results of the present study are in line with the open-label study conducted by Raut et al. on 18 healthy volunteers who received increasing doses of Ashwagandha extract for 30 days to evaluate its effect on muscle strength and ascertain its safety. The findings demonstrated that, in greater dosages (from 750 to 1,250 mg), Ashwagandha extract had no negative effects on the hematological and biochemical organ function and had positive effects on muscle strength [9].

Tripathi et al. conducted an open-label, placebo-controlled pilot study on 30 healthy individuals who had received 330 mg and 500 mg of aqueous extract of Ashwagandha. The study evaluated the effect of Ashwagandha extract on physical performance and cardiovascular parameters during exercise. The study highlighted that, on day 28, both the groups showed a significant increase in mean distance travelled and average speed compared to placebo (p<0.005). In comparison to the placebo group, the Ashwagandha extract groups significantly reduced mean SBP during fixed workload exercise (p<0.05) [10]. Ziegenfuss et al. conducted a 12-week double-blind, placebo-controlled (7.5 km cycling time) trial with an aqueous extract of the roots and leaves of WS on 38 recreationally active men to assess body composition, muscular strength, power, and endurance. All aerobic endurance variables and body composition showed statistically significant improvement in the WS-supplemented group but not in the placebo group [29]. Similar findings were also revealed in other clinical studies conducted by Wankhede et al. [30] and systematic review by Bonilla et al. [31].

These pharmacological effects of Ashwagandha are attributed to its *balyavardhak *properties and are in sync with specific pharmacodynamic effects as explained in Ayurvedic principles of Dravyagunavidnyan (Ayurvedic pharmacology) [32,33]. The primary focus of the current study has been on ‘mamsavivardhan’ (enhancing muscle mass and function), and the results of this study corroborate with this classical Ayurveda indication. Scientists have attempted to understand the molecular mechanisms of muscle activity through experimental models [34,35 ]. However, whether the muscle activity can be attributed to an identified phytomolecule or it is a collective effect of several phytoconstituents remains to be unraveled.

Currently, no clinical studies have evaluated the effect of WS on muscle status biomarkers. The present study noted that, at day 60, the inflammatory biomarkers hs-CRP, interleukin-6, TNF-α, and serum myostatin, a marker of muscle wasting, exhibited a decreasing trend in the Ashwagandha extract group, whereas the markers rose in the placebo group, particularly hs-CRP and lactate dehydrogenase. Myostatin, a myokine protein released by skeletal muscle and a member of the TGF superfamily, functions as a regulator of the skeletal muscle-formation process. Myostatin inhibition helps decrease fat storage and boosts muscle growth and strength [36]. Therefore, a gradual fall in these biomarkers at the follow-up visits points out the utility of Ashwagandha extract as a potential candidate for treating conditions associated with muscle wasting.

In the digit-substitution test and choice-discrimination test, the reaction time showed a statistically significant reduction for the Ashwagandha extract group, which was not seen in the placebo group. This is suggestive of improved alertness in the Ashwagandha extract group, which has been reported to improve mental alertness on awakening [9,13].

There was no significant adverse event reported from both the groups. The laboratory investigations for haematological and serological profiles were within normal limits before and after intervention in both groups, confirming the safety of Ashwagandha extract in a given dose for the duration of 60 days. However, both groups saw a significant increase in blood cholesterol and an increase in S. triglycerides on day 60. This may be supported by the fact that the healthy volunteers of the research, although at the baseline, were fasting for 12 hours before the collection of blood; at the end of the study, they fasted for just eight hours.

The study did have limitations as the duration of the study was limited to 60 days. Further longer duration studies in individuals having sarcopenia of different origins are desirable to fully elucidate the potential of Ashwagandha. The current study's generalizability is limited by the inclusion of only healthy male volunteers. Further, formal sample size calculation was not done to estimate the change in biomarkers.

## Conclusions

Ashwagandha (W. somnifera), is a promising candidate to enhance physical performance, improve skeletal muscle mass and muscle function, down-regulate inflammation, and prevent muscle wasting. It was found to be safe in healthy individuals as evidenced by clinical profile, laboratory investigations, and test of alertness. Ashwagandha appears to be a potential candidate that can be developed as a product for treating sarcopenia. Hence, it is desired to further investigate the potential of Ashwagandha in robust clinical trials.
